# *Perna canaliculus* Lipid Complex PCSO-524™ Demonstrated Pain Relief for Osteoarthritis Patients Benchmarked against Fish Oil, a Randomized Trial, without Placebo Control

**DOI:** 10.3390/md11061920

**Published:** 2013-06-05

**Authors:** Marek Zawadzki, Claudia Janosch, Jacek Szechinski

**Affiliations:** Clinic of Rheumatology and Internal Medicine, Academic Clinical Hospital, Wroclaw 50-556, Poland; E-Mails: mareklek.9@wp.pl (M.Z.); jacek.szechinski@gmail.com (J.S.)

**Keywords:** *Perna canaliculus*, PCSO-524™; Lyprinol^®^, greenlipped mussel, osteoarthritis, arthritis, fish oil, pain relief, joint pain

## Abstract

Osteoarthritis (OA) typically generates pain, reduced mobility and reduced quality of life. Most conventional treatments for osteoarthritis, such as non-steroidal anti-inflammatory drugs (NSAIDs) and simple analgesics, have side effects. PCSO-524™, a non polar lipid extract from the New Zealand Green Lipped Mussel, is rich in omega-3 fatty acids and has been shown to reduce inflammation in both animal studies and patient trials. This OA trial examined pain relief changes in relation to quality of life and safety of use for OA patients who took PCSO-524™ compared with patients who took fish oil (containing an industry standard EPA-18% and DHA-12% blend). PCSO-524™ patients showed a statistically significant improvement compared with patients who took fish oil. There was an 89% decrease in their pain symptoms and 91% reported an improved quality of life. Patients treated with fish oil showed significantly less improvement and a greater level of physical discomfort during the study. These results suggest that PCSO-524™ might offer a potential alternative complementary therapy with no side effects for OA patients.

## 1. Introduction

Since the 1980s, numerous clinical studies have shown that both the oil of *Perna canaliculus* (PCSO-524™) and 18/12 fish oil, standardised to contain 18% eicosapentaenoic (EPA) and 12% docosahexaenoic acids (DHA), have anti-inflammatory activity that can contribute to reduced pain and improved joint mobility for patients who suffer osteoarthritis (OA) [1,2,3]. PCSO-524™ has unique polyunsaturated fatty acids (PUFAs) [4,5]; including 5,9,12,15-octodecatetraenoic acid (OTA), 5,9,12,16-nonadecatertraenoic acid, 7,11,14,17-eicosatetraenoic acid (ETA), and 5,9,12,15,18-heneicosapentaenoic acid [4]. These molecules (including EPA and DHA) are similar to arachidonic acid (AA) (5,8,11,14-eicosatetraenoic acid), the precursor to the inflammatory agents, prostaglandins and leukotrienes. In case of ETA the first double bond is located at the seventh position, and the second double bond is interrupted from the first by two methylene groups, resulting in the double bonds at positions 7, 11, 14 and 17. A similar pattern is shown for the three other novel compounds, whereby the second double bond is separated from the first by more than one methylene group. The interrupted bond positioning of these structural analogues of AA may account for their anti-inflammatory (AI) behaviour, by competitively inhibiting the active site of enzymes that use AA as a substrate [5,6]. This combination of omega-3 fatty acids is not found in any other known marine oils [4]. Furthermore, fish oil studies typically use large dosages of standardised EPA and DHA fish oil [7,8] compared with similar studies [9,10] that used the oil of *P. canaliculus* to achieve reductions in inflammatory markers.

Former studies [11,12] had compared the lipid fraction of *P. canaliculus* with a freeze-dried preparation and demonstrated significant improvements in the AI effect of the extracts of *P. canaliculus* due to a new stabilisation production process. Further research compared both the stabilised extract powder and the stabilised oil (now trademarked as PCSO-524™ and available commercially as Lyprinol^®^ and OmegaXL^®^) and confirmed the effectiveness of the stabilised extract [13,14] to potentially provide physicians with an additional complementary therapy for the treatment of symptoms associated with OA and rheumatoid arthritis (RA).

Numerous fish oil studies over the years have also shown reduced inflammatory responses [15,16]. These studies covered asthma, exercise-induced inflammation and arthritis. Positive results usually require large doses of up to 20 g of fish oil to achieve a therapeutic dose [14,16]. It is well known that people should use fish oil under medical supervision if they bruise easily, have a bleeding disorder, or are also using anticoagulants. Large doses of omega-3 fatty acids may increase the risk of bleeding and can cause stomach gas, bloating, belching and diarrhoea.

In the randomised double-blind, clinical trial performed by Szechinski and co-worker [17], OA patients were administered with either PCSO-524™ (Group A) or standardised 18/12 fish oil capsules (Group B) for 12 weeks. A statistically significant improvement of both their pain symptoms related to OA and quality of life could be demonstrated (first stage of the study). The second stage provided an opportunity for the participants who had been administered 18/12 fish oil to also repeat the 12-week study taking PCSO-524™, the results of which are labelled under Group C. The results of the second stage are presented here. The significance of the study is comparative. Patients often use well-known analgesics such as paracetamol or ibuprofen to treat pain related to OA. Long-term intake of these analgesics may cause gastro-intestinal side effects and might also adversely affect the kidneys. PCSO-524™ has been shown to be an effective complementary or alternative active ingredient for the treatment of OA without any side effects [3].

One of the objectives of the study was to ascertain if a similar dose of PCSO-524™ (400 mg) can achieve the same AI benefits as larger doses of standardised fish oil (1200 mg). If so this would provide practitioners with a safer first-line alternative that does not carry the risk of haemorrhage. It may also enable a reduction of intake of analgesics.

## 2. Experimental Section

The procedures followed here, were in accordance with the Helsinki Declaration of 1975, as revised in 2000. The clinical trial was supervised by the Department of Public Health of the Wroclaw Medical University Hospital, Poland. At the time of conducting this clinical trial, which was using a food ingredient, it was not necessary to be registered and be supervised by an external ethics committee.

### 2.1. Patients

#### 2.1.1. Characteristics of the Groups of Patients Involved in the Study

The patients were recruited from the outpatient clinic of the Wroclaw Medical University Hospital, Poland. The inclusion criteria were restricted to clinically diagnosed knee and/or hip osteoarthritis patients older than 50 years and who were not taking prescription anti-inflammatory medication. Both genders were accepted. The included patients also had radiographic evidence of osteophytes, or confirmation by X-ray images. Patients who had concurrent rheumatoid arthritis, or other connective-tissue disorders, patients who were using prescription medication, or who were being treated with anticoagulants were excluded from the trial.

**Figure 1:**
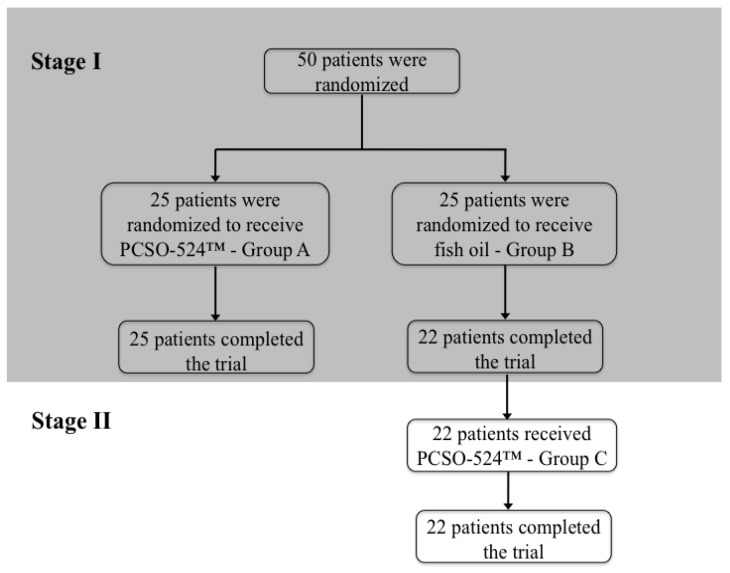
Study flow chart.

Initially, there were 50 patients (44 women and 6 men) in the study group. Twenty-five patients were assigned randomly into one of Group A or Group B (stage I, Figure 1).

All 25 patients from Group A (PCSO-524™) completed the treatment. Only 22 patients from Group B (fish oil) completed the treatment. Two patients from Group B were excluded because of adverse effects of the fish oil (diarrhea, nausea, stomach aches, increased arterial blood pressure). One patient was excluded due to vacation commitments. After they finished stage I, all 22 patients from Group B continued the study under the same protocol as the PCSO-524™ Group A patients and then formed Group C (stage II). All patients completed the second stage (Figure 1).

The patient characteristics, which compared all groups at baseline, did not show a statistically significant difference with regards to age, gender, weight, height, BMI, systolic blood pressure (SBP) and diastolic blood pressure (DBP) (Table 1, Table 2, SD values are given in brackets).

**Table 1:** Characteristics of patients at the baseline.

Features	A (PCSO-524™; Stage I) Mean (SD)	B (Fish oil; Stage I) Mean (SD)	C (PCSO-524™; Stage II) Mean (SD)
Total No. of patients	25	25	22
Age [year]	65.58 (±9.49)	66.72 (±8.42)	67.23 (±9.06)
Gender [F/M]	22/3	22/3	19/3
Weight [kg]	78.20 (±15.45)	75.70 (±11.80)	78.39 (±14.63)
Height [m]	1.65 (±0.07)	1.64 (±0.08)	1.63 (±0.08)
BMI	28.68 (±5.76)	28.25 (±4.49)	29.53 (±5.48)
SBP [mmHg]	130.40 (±12.90)	131.40 (±17.23)	134.54 (±15.65)
DBP [mmHg]	78.00 (±9.78)	79.20 (±9.42)	78.64 (±7.10)

Abbreviations: BMI = body mass index; SBP = systolic blood pressure; DBP = diastolic blood pressure.

**Table 2:** Laboratory characteristics of patients at the baseline.

Features	A (PCSO-524™; Stage I) Mean (SD)	B (Fish oil; Stage I) Mean (SD)	C (PCSO-524™; Stage II) Mean (SD)
WBC (×10^9^/L)	5.87 (±1.18)	7.02 (±2.60)	6.52 (±1.01)
RBC (×10^9^/L)	4.45 (±0.24)	4.50 (±0.37)	4.50 (±0.42)
Hgb (g/dL)	13.29 (±0.83)	13.37 (±0.98)	12.98 (±1.57)
Ht (L/L)	39.94 (±1.75)	40.89 (±2.84)	39.66 (±4.14)
PLT (×10^9^/L)	265.64 (±76.93)	255.91 (±97.37)	287.05 (±102.95)
Aspat (u/L)	20.82 (±6.93)	23.50 (±7.98)	25.23 (±19.14)
Alat (u/L)	19.41 (±8.21)	19.88 (±7.57)	24.19 (±23.61)
ESR (mm/h)	25.35 (±22.35)	18.15 (±13.15)	19.20 (±12.33)

Abbreviations: WBC = white blood cell count; RBC = red blood cell count; Hgb = haemoglobin; Ht = haematocrit; Plt = platelet count; Alat = alanine aminotransferase; Aspat = aspartate aminotransferase; ESR = erythrocyte sedimentation rate.

In stage I, there was neither a bias in the type of afflicted joint nor in the type of joint pain in either of the groups. In stage II almost all people showed joint involvement in both knees, whereas in Groups A and B only half of the patients showed joint involvement at this location (Table 3, Table 4). Each subject commenced with a baseline assessment followed by four separate assessment sessions with the physician during a three-month period. The study was conducted during a five-month period in total.

The disease durations were between four to 14 years in Group A, and between two to 16 years in Groups B and C.

**Table 3:** Comparative characteristics of osteoarthritis (OA) patients by the type of afflicted joint at base line.

Joints involvement	A (PCSO-524™; Stage I)	B (Fish oil; Stage I)	C (PCSO-524™; Stage II)
Total No. of patients	25	25	22
Both knees	13 (52.0%)	14 (56.0%)	14 (63.6%)
Hip joints	4 (16.0%)	2 (8.0%)	0 (0.0%)
Both knees and hip joints	0 (0.0%)	1 (4.0%)	1 (4.5%)
Hip joint, spinal joints	1 (4.0%)	0 (0.0%)	0 (0.0%)
Nonspecific	7 (25.0%)	8 (32.0%)	7 (31.8%)

**Table 4:** Comparative characteristics of OA patients by joint pain type at base line.

Joint pain location	A (PCSO-524™; Stage I)	B (Fish oil; Stage I)	C (PCSO-524™; Stage II)
Total No. of patients	25	25	22
Knee joints	17 (68.0%)	18 (68.0%)	16 (72.7%)
Knee and hip joints	1 (4.0%)	1 (4.0%)	0 (0.0%)
Nonspecific	7 (28.0%)	6 (28.0%)	6 (27.3%)

#### 2.1.2. Blinding and Randomization

Randomization was obtained by coded labeling of packs, performed by a person independent of the study. Both preparations were filled into 150 mg capsules that had similar appearance and odour. In stage I, the study was blind and the patients were randomly divided into Group A and Group B. The physicians gave the capsules and the dose instructions to the patients in packages. The current treatment was not changed and no other drugs, except paracetamol as rescue medication, were introduced during the three-month study period for each subject.

In stage II, the patients who were in Group B were advised that they had been treated previously with fish oil and could participate further in stage II and be treated with PCSO-524™, as was done for Group A in stage I. This second stage of the study was not blind, because both physicians and patients knew that PCSO-524™ would be administered. The dose instructions and the rescue medication were the same as during stage I.

#### 2.1.3. Intervention Treatment

During a period of 12 weeks, the interventions were a dosage of four capsules, taken twice per day and thus 1200 mg of preparation per day.

Due to the known side effects of fish oil and the fact that our patients were elderly, we decided to conduct this preliminary study using smaller dosages of fish oil with comparable intake as would be prescribed for PCSO-524™.

Lyprinol^®^ (150 mg/capsule) contained 50 mg of patented CO_2_ extracted greenshell mussel non-polar lipids (PCSO-524™), 99.85 mg of pharmaceutical-grade olive oil and 0.15 mg natural vitamin E as a stabilizer (total omega-3 5.2% EPA and 3.4% DHA).

Fish oil (150 mg/capsule) contained 150 mg of fish oil (total omega-3 18% EPA and 12% DHA) produced by Ocean Nutrition.

### 2.2. Study Design

#### 2.2.1. Visual Analog Scale (VAS) for Patient Self-Assessment of Pain

The patients provided an assessment of their OA pain level through the use of the 100 mm visual analogue scale, which was reviewed by the physician in three four-week intervals.

#### 2.2.2. Health Assessment Questionnaire (HAQ) for Patient Self-Assessment of Activity

This questionnaire was designed originally for patients who had inflammatory joint disorders. The HAQ consists of 20 questions, which cover eight categories of daily physical activities. These included dressing, arising, hygiene, walking, heating, eating, grip and daily activities. Questions could be answered with “possible without any difficulty (0 point)”, “possible with some difficulty (1 point)”, “possible with much difficulty (2 points)” and “unable to do (3 points)”. Use of any aids increased the score by 1 point. The worst score of each category was accepted as representative value for this activity. The total HAQ score was equal to the median value of all sub scores. The physician reviewed this questionnaire at four-week intervals. A reduction of a HAQ value ≥ 0.25 was evaluated as a significant clinical improvement. The improvement of the level of physical activities was considered to be an improvement of quality of life.

#### 2.2.3. Improvement of Health and Disease Condition

Patients had to draw pictures in their personal assessment journals.

#### 2.2.4. Safety Assessment

At the baseline and at the end of the study, the analytical testing included blood counts (white blood cells, red blood cells, haemoglobin, haematocrit, platelet count, liver function testing (Aspat, Alat) and erythrocyte sedimentation rate (ESR).

#### 2.2.5. Tolerance of the Intervention Treatments

At each of the three four-week assessments, a physician enquired about adverse reactions to intervention treatment.

**Table 5:** Summary of the visual analog scale (VAS).

Assessment		Group A (PCSO-524™; stage I)				Group B (Fish oil; stage I)				Group C (PCSO-524™; stage II)			
		week				week				week			
		0	4	8	12	0	4	8	12	0	4	8	12
Pain	mean/median *	66.00	31.56	8.00	1.00	66.39	65.45	64.91	69.00	56.00	22.50	8.00	1.00
	SD/IQR **	11.48	12.46	9.00	3.00	16.01	15.89	16.26	30.00	25.00	28.00	9.00	3.00
	min	/	/	0.00	0.00	/	/	/	35.00	15.00	8.00	0.00	0.00
	max	/	/	55.00	61.00	/	/	/	80.00	92.00	77.00	55.00	61.00
	distribution	nor	nor	nnor	nnor	nor	nor	nor	nnor	nnor	nnor	nnor	nnor
Disease activity	mean/median *	62.64	25.04	6.00	1.00	62.57	62.64	59.67	60.00	56.09	27.64	11.50	6.00
	SD/IQR **	13.15	10.17	8.00	3.00	15.54	16.34	15.70	33.00	18.30	18.31	7.00	6.00
	min	/	/	0.00	0.00	/	/	/	31.00	/	/	3.00	3.00
	max	/	/	56.00	54.00	/	/	/	79.00	/	/	74.00	55.00
	distribution	nor	nor	nnor	nnor	nor	nor	nor	nnor	nor	nor	nnor	nnor
General health	mean/median *	62.96	25.52	13.00	2.00	61.00	62.68	61.10	65.00	54.00	33.00	17.00	9.50
	SD/IQR **	12.57	11.29	11.00	4.00	38.00	14.63	15.13	35.00	20.00	30.00	12.00	8.00
	min	/	/	9.00	0.00	35.00	/	/	34.00	8.00	8.00	4.00	5.00
	max	/	/	0.00	53.00	89.00	/	/	83.00	9.00	70.00	64.00	50.00
	distribution	nor	nor	nnor	nnor	nnor	nor	nor	nnor	nnor	nnor	nnor	nnor

*—in nor, median in nnor distribution; **—SD in nor, IQR in nnor distribution; nor = normally (parametrically); nnor = non-parametrically; *p*-Values: Pain: A *vs.* B = significant; B *vs.* C = significant; A *vs.* C = not significant; Disease activity: A *vs.* B = significant; B *vs.* C = not significant; A *vs.* C = not significant; General health: A *vs.* B = significant; B *vs.* C = significant; A *vs.* C = not significant; VAS Pain scoring: 100–80: agonizing to horrible; 80–60: horrible to dreadful; 60–40: dreadful to uncomfortable; 40–20: uncomfortable to annoying; 20–0: annoying to none.

**Table 6:** Summary of the health assessment questionnaire (HAQ).

Feature	Group	Week																Significance *p*
		Baseline				4				8				12				
		Median	IQR	Min	Max	Median	IQR	Min	Max	Median	IQR	Min	Max	Median	IQR	Min	Max	
Dressing	A	0.00	0.50	0.00	1.50	0.00	0.00	0.00	1.00	0.00	0.00	0.00	0.00	0.00	0.00	0.00	0.00	<0.05
	B	0.50	0.75	0.00	2.00	0.00	1.00	0.00	2.00	0.00	1.00	0.00	2.00	0.00	1.00	0.00	2.00	ns
	C	0.50	1.00	0.00	2.00	0.00	0.50	0.00	2.00	0.00	0.00	0.00	2.00	0.00	0.00	0.00	2.00	<0.05
Arising	A	0.50	1.00	0.00	2.00	0.00	0.50	0.00	1.50	0.00	0.00	0.00	1.50	0.00	0.00	0.00	1.50	<0.05
	B	0.75	1.00	0.00	2.00	0.50	1.00	0.00	1.50	1.00	1.00	0.00	1.50	1.00	1.00	0.00	2.00	ns
	C	0.50	1.00	0.00	2.00	0.00	1.00	0.00	2.00	0.00	0.00	0.00	2.00	0.00	0.00	0.00	1.00	<0.05
Eating	A	0.33	0.67	0.00	1.33	0.00	0.33	0.00	0.67	0.00	0.00	0.00	0.67	0.00	0.00	0.00	0.67	<0.05
	B	0.00	0.50	0.00	1.33	0.00	0.67	0.00	1.33	0.00	0.33	0.00	1.67	0.00	0.33	0.00	1.67	ns
	C	0.17	0.67	0.00	1.33	0.00	0.00	0.00	1.67	0.00	0.00	0.00	1.00	0.00	0.00	0.00	0.00	<0.05
Walking	A	0.50	0.50	0.00	2.00	0.00	0.50	0.00	1.50	0.00	0.00	0.00	1.00	0.00	0.00	0.00	1.00	<0.05
	B	0.50	1.00	0.00	2.00	0.50	1.00	0.00	2.00	0.50	1.00	0.00	2.00	1.00	1.00	0.00	2.00	ns
	C	0.25	1.00	0.00	2.00	0.00	0.50	0.00	2.00	0.00	0.00	0.00	2.00	0.00	0.00	0.00	2.00	<0.05
Hygiene	A	0.33	0.50	0.00	1.67	0.50	0.00	0.00	1.50	0.00	0.00	0.00	0.67	0.00	0.00	0.00	0.67	<0.05
	B	0.33	1.00	0.00	1.67	1.00	0.67	0.00	2.00	0.33	1.00	0.00	2.00	0.33	1.00	0.00	2.00	ns
	C	0.33	0.00	0.00	1.33	1.00	0.67	0.00	3.00	0.00	0.00	0.00	1.33	0.00	0.00	0.00	1.00	<0.05
Heating	A	1.00	0.50	0.00	2.00	0.50	0.50	0.00	1.50	0.50	0.50	0.00	1.00	0.00	0.50	0.00	1.00	<0.05
	B	1.00	1.00	0.00	3.00	1.00	1.00	0.00	2.00	1.00	1.00	0.00	3.00	1.00	0.50	0.00	3.00	ns
	C	1.00	0.00	0.00	3.00	1.00	1.00	0.00	3.00	0.00	0.50	0.00	3.00	0.00	0.00	0.00	2.50	<0.05
Grip	A	0.00	0.33	0.00	1.00	0.00	0.00	0.00	1.00	0.00	0.00	0.00	0.67	0.00	0.00	0.00	0.33	<0.05
	B	0.17	0.67	0.00	1.33	0.17	1.00	0.00	1.00	0.17	1.00	0.00	1.33	0.33	1.00	0.00	1.33	ns
	C	0.17	0.67	0.00	2.00	0.00	0.33	0.00	2.00	0.00	0.00	0.00	2.33	0.00	0.00	0.00	2.00	<0.05
Daily activities	A	0.67	0.67	0.00	2.00	0.33	0.33	0.00	1.00	0.00	0.33	0.00	1.00	0.00	0.00	0.00	1.00	<0.05
	B	0.83	0.67	0.00	2.67	1.00	0.67	0.00	1.67	1.00	0.67	0.00	3.00	1.00	1.00	0.00	3.00	ns
	C	0.67	0.67	0.00	2.67	0.67	1.00	0.00	2.67	0.33	0.67	0.00	2.67	0.00	0.33	0.00	2.67	<0.05
HAQ (total)	A	0.42	0.44	0.00	1.38	0.21	0.31	0.00	0.85	0.06	0.21	0.00	0.54	0.00	0.06	0.00	0.54	<0.05
	B	0.42	0.79	0.00	1.85	0.39	0.52	0.00	1.52	0.64	0.79	0.00	1.88	0.54	0.69	0.00	1.88	ns
	C	0.41	0.79	0.00	1.90	0.21	0.52	0.00	2.13	0.07	0.17	0.00	2.04	0.00	0.13	0.00	1.65	<0.05

According to the distribution test analyses, all values within the groups and features were non-parametrically distributed; ns = not significant.

#### 2.2.6. Statistical Analysis

The randomized codes were broken by a statistician and the results were analyzed independently by a medical statistician to determine their statistical significance. The analyses of differences between two groups were performed by the Mann-Whitney U test in case of non-parametric distribution, and by the Student’s t-test in case of parametric distribution. The differences for variables with regard to duration of treatment were calculated by the Kruskall-Wallis ANOVA test for non-parametric distribution variables and ANOVA in parametric distribution variables. The differences in qualitative variables were analyzed by the chi-squared test and by the differences between a two-structure-indicators test. Distribution test analysis was performed using: (W) Shapiro-Wilk and Kolmogorov Smirnov test. In case of normal distribution, results are presented as mean values with ±SD (Table 1, Table 2). In case of non-normal distribution, results are presented as median values with IQR (Table 5, Table 6).

## 3. Results

The results presented here are a review (Groups A and B) with appended results (Group C) of the Szechinski study [17].

### 3.1. Patients

At the end of stage 1 of the study, all 25 patients from Group A (PCSO-524™) completed the treatment. Only 22 patients from Group B (fish oil) completed the treatment. Three patients from Group B were excluded because of adverse effects of the fish oil (diarrhoea, nausea, stomach aches, increased arterial blood pressure). In stage II, all 22 patients completed the trial.

### 3.2. Efficacy Assessment

The rescue medication, paracetamol, was used by nine patients from Group A, 13 patients from Group B and four patients from Group C to decrease pain and/or discomfort during the study. More patients taking fish oil required the rescue medication than those taking PCSO-524™.

#### 3.2.1. Visual Analogue Scale (VAS)

**Figure 2:**
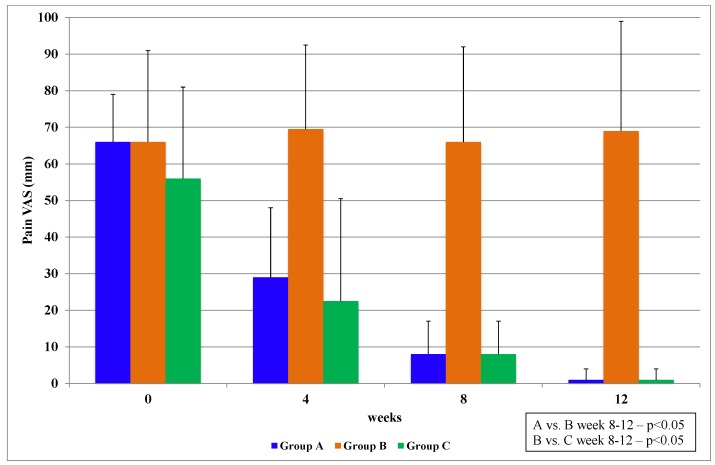
Baseline VAS for pain score and changes in the VAS score of the groups of patients studied. Group A = PCSO-524™; Stage I; Group B = fish oil; Stage I; Group C = PCSO-524™; Stage II. Results are expressed as median values with IQR.

The patient assessment pain scores for intervention treatments showed a significant improvement for PCSO-524™ within four weeks (Groups A and C). The longer the period of administration of PCSO-524™ the greater the relief from pain and stiffness associated with the symptoms of OA. Patients who were administered fish oil did not show any improvements that were statistically significant within the same period (Figure 2; Table 5).

#### 3.2.2. Health Assessment Questionnaire (HAQ) Index

**Figure 3:**
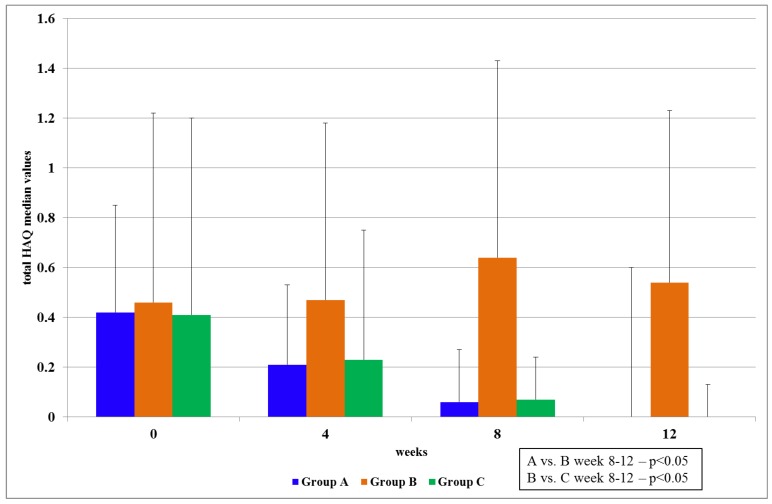
HAQ total score Group A = PCSO-524™; Stage I; Group B = fish oil; Stage I; Group C = PCSO-524™; Stage II. Results are expressed as median values with IQR.

An improvement was observed in all the categories of the index for patients who were administered PCSO-524™ (Groups A and C; Figure 3; Table 6).

#### 3.2.3. Improvement of Health and Disease Condition

A significant improvement of overall health condition was observed in 88% of Group A patients. In contrast, 59% of patients from Group B showed improvement (Table 7). Two Group A patients and one Group B patient could not clearly define any change or improvement.

**Table 7:** Improvement after treatment.

Improvement after treatment	A (PCSO-524™; Stage I)	B (Fish oil; Stage I)	C (PCSO-524™; Stage II)
Total No. of patients	25	22	22
Yes	22 (88.00%)	13 (59.09%)	21 (95.45%)
No	1 (4.00%)	8 (36.36%)	1 (4.55%)
No defined change	2 (8.00%)	1 (4.55%)	0 (0.00%)

The Pearson’s chi-square test value of all 3 groups was 7.04 (*p* = 0.13).

In Group C, 95% of the patients showed an improvement with the intake of PCSO-524™. In summary, 87% of the patients treated with PCSO-524™ confirmed improvements of their OA symptoms.

#### 3.2.4. Safety Assessment

There were no statistically significant changes to blood parameters, liver function tests or erythrocyte sedimentation rates during the study. Patients who took PCSO-524™ showed a trend of improvement in their blood-cell count (increase of haemoglobin, haematocrit and erythrocytes; data not shown).

#### 3.2.5. Tolerance

In stage I, all 25 patients in Group A reported 100% tolerance of the treatment without any side effects. In contrast, seven patients from Group B reported adverse side effects from the fish oil during the four-week assessments. Two females of these seven patients withdrew from the study (bad tolerance: diarrhea, stomach ache, increased arterial blood pressure) and four patients reported side effects: nausea during the first day; constipation and stomach ache during the first month; headaches, pain in the kidney area; frequent pain in the liver area. One female patient withdrew for a vacation (Table 8).

In stage II, three patients of Group C also reported some minor side effects (nausea during the first day, headaches) during the intake of PCSO-524™ (Table 8).

**Table 8:** Tolerance of treatment.

Tolerance	A (PCSO-524™; Stage I)	B (Fish oil; Stage I)	C (PCSO-524™; Stage II)
Total No. of patients	25	24	22
Good	25 (100.00%)	17 (70.83%)	19 (86.36%)
Generally good	0 (0.00%)	4 (16.67%)	3 (13.64%)
Bad	0 (0.00%)	3 (12.50%)	0 (0.00%)

The Pearson’s chi-square test value of all 3 groups was 12.61 (*p* = 0.002).

## 4. Discussion

The aim of this clinical trial was to evaluate the comparative effectiveness of PCSO-524™ relative to fish oil for pain relief, quality of life and safety.

In stage I, the patients from Group A, who were treated with PCSO-524™, showed a statistically significant reduction of pain, improved levels of mobility and activity and 100% tolerance with no noted side effects. The efficacy results for PCSO-524™ oil are similar to those reported in other studies [3,11,12,13,18,19,20]. In comparison, patients from Group B, who were treated with fish oil, did not show a notable reduction in pain, there was no significant improvement of mobility or activity and only 71% tolerance. In stage II, the patients knew that they had taken PCSO-524™ oil and those patients also showed a reduction of pain. However, due to the not-blinded part of the study, these results are consequently less valuable. Furthermore, neither stage was placebo-controlled (e.g., olive oil). Other PCSO-524™ studies have already documented the comparative results of placebo-controlled trials [3].

PCSO-524™ is produced from the New Zealand green lipped mussel, a common species found in the protected waters around New Zealand. The non-polar lipids are removed from the mussel through the use of a patented CO_2_ supercritical extraction process. PCSO-524™ is a very complex mixture of lipid classes which are high in free fatty acids. PCSO-524™ was compared with fish oil, which was found to be more effective in inhibition of the cyclooxygenase (COX) pathway [6]. COXs are lipid metabolizing enzymes that catalyze the oxygenation of PUFA, usually AA, to form the prostanoids, which are potent cell-signaling molecules associated with the initiation, maintenance and resolution of inflammatory processes [21].

Fish oil is composed predominantly of triglyceride molecules that are rich in EPA and DHA, while PCSO-524™ is more complex with more than 60 lipid compounds. Seemingly, fish oil results in the release of inhibitory PUFAs that give it similar activity in the anti-inflammatory pathways, but with less efficacy than PCSO-524™ [6]. In a preliminary toxicology assessment, it was shown that the CO_2_ lipid extract and its free fatty acid (FFA) components caused biologically significant AI activity *in vivo*, with no apparent adverse side effects [6]. In a more detailed analysis, the FFA class was fractionated from the CO_2_ lipid extract and the FFA components demonstrated inhibition of inflammatory markers [5]. As already mentioned, the new structurally-related family of omega-3 PUFAs that was identified included C18:4, C19:4, C20:4, and C21:5 PUFA in the fractions with high anti-inflammatory effect. The C20:4 was the predominant PUFA in the *P. canaliculus* lipids and was identified as a structural isomer that mimicked AA. The novel anti-inflammatory compounds are understood to compete much more efficiently than other pro-inflammatory fatty acids for the COX and lipoxygenase (LOX) pathways [4]. The latter pathway is responsible for the transformation of AA in the cell membrane into leukotrienes and the mechanism of AI activity of PCSO-524™ has been analyzed before [5]. It prevented the migration of neutrophils and alleviated the signs of OA, such as pain, swelling and stiffness. Taking PCSO-524™ to relieve pain is a process. Typically, the AI effect starts to be noted within four to eight weeks to reduce the symptoms of chronic inflammation.

As the fish oil capsules contain a standardized amount of EPA (18%) and DHA (12%), we did not expect to observe such a major difference between patients treated with PCSO-524™ (Lyprinol^®^: 5.2% EPA and 3.4% DHA) and patients treated with fish oil. This result requires further research regarding the active substances in the two intervention treatments and the dosage levels.

Interestingly, OA patients who took PCSO-524™ (Group A and Group C) had a notable reduction of clinical symptoms in the first four weeks and a continuous improvement during the 12 weeks of the study. This was in line with previous findings [9] that the lipid fractions of *P. canaliculus* could achieve significant clinical improvements in the first four weeks in some patients who had RA.

Other studies support the view that dietary omega-3 PUFA, particularly EPA and DHA, are important modulators of a host’s inflammatory/immune responses [22,23]. It has been reported that large dosages of omega-3 PUFA are probably needed during long-duration therapy for diseases characterized by immune dysfunction [23]. Olveira and co-workers established results with fish oil after 12 months in patients who had cystic fibrosis [22]. Our study demonstrated that EPA and DHA in PCSO-524™ alone are not responsible for the AI effects, which recognizes previous studies regarding PCSO-524™ and EPA [13] in rats and COX inhibition analyses of PUFAs of PCSO-524™ [19]. Indeed, in another study it could be demonstrated that furan fatty acids (F-acids) also play an important AI role in PCSO-524™ [24]. However, this needs further investigations. 

Additionally, the *P. canaliculus* oil certainly showed that a quicker and more observable reduction in pain could be achieved with smaller dosages. There were no statistically significant results from fish oil for the administered dosage during the 12 weeks. This could have been due to the dosage of fish oil and also to the short duration of the trial, but larger dosages of fish oil for elderly patients would generally not be recommended.

Furthermore, it was noteworthy that patients from the PCSO-524™ group had less demand for a supportive treatment with paracetamol during the study (data not shown). Only six patients (36%) from Group A and four patients (18%) from Group C decided to use additional treatment with paracetamol, while in Group B 64.64% of the patients used additional paracetamol treatment to alleviate pain.

## 5. Conclusions and Outlook

Stabilized *P. canaliculus* oil has become a well-known natural inhibitor of COX and LOX, the activity of which has been referenced in numerous clinical trials, with no adverse effects.

The patients from Groups A and C judged the efficacy of PCSO-524™ positively with regard to pain relief within the first four weeks of administration and considered it to be beneficial for their quality of life. In both Groups A and C, patients continued to show further reductions in pain during the 12-week period of the study. The benefits of fish oil were not evident during this 12-week trial. The reasons for this conclusion included the side effects, large dosages needed and the duration required for fish oil to have an effect. Practitioners, from these results could consider stabilized *P. canaliculus* oil as a safer and faster-acting alternative complementary therapy for patients who suffer from OA compared with the use of fish oil.

In the future, clinical trials should always be placebo-controlled and perform a deeper examination of the dosage of PCSO-524™. Could higher dosages lead to a quicker pain relief for OA patients without any side effect? In addition, biochemical analyses should have a closer investigation to the molecules, which leads to the AI effect of PCSO-524™, because EPA and DHA are not (alone) responsible for it.
